# Minimally Invasive Segmental Scleral Buckle as Primary Surgery for the Repair of Rhegmatogenous Retinal Detachment: A Descriptive Case Series Study

**DOI:** 10.1155/crop/2135418

**Published:** 2026-07-24

**Authors:** Supanji Supanji, Dewi Fathin Romdhoniyyah, Hifdza Faza Felisha, Felicia Widyaputri, Tiara Putri Utami, Erin Arsianti, Mohammad Eko Prayogo, Firman Setya Wardhana, Muhammad Bayu Sasongko

**Affiliations:** ^1^ Department of Ophthalmology, Faculty of Medicine Public Health and Nursing, Universitas Gadjah Mada, Yogyakarta, Indonesia, ugm.ac.id; ^2^ Dr. Sardjito General Hospital, Yogyakarta, Indonesia; ^3^ Ophthalmology Clinic, Dr. YAP Eye Hospital, Yogyakarta, Indonesia

**Keywords:** case series, outcome, rhegmatogenous retinal detachment, RRD, segmental scleral buckle, SSB

## Abstract

**Introduction:**

The scleral buckling (SB) or encircling buckle technique has been employed in the management of rhegmatogenous retinal detachment (RRD). While encircling buckle technique offers substantial assistance in managing RRD, particularly for cases involving numerous breaks or extensive detachments, this procedure is time‐consuming and associated with complications such as anterior segment ischemia. In contrast, segmental scleral buckling (SSB) provides a more targeted approach for correcting retinal detachments. Thus, SSB is technically straightforward in the hands of an experienced vitreoretinal surgeon. Herein, we delineate anatomical and functional results of SSB in RRD patients.

**Result:**

Seven consecutive patients (seven eyes) with primary RRD were treated with the SSB technique and followed up for 6 months. Postoperatively, all seven individuals exhibited satisfactory buckle positioning and height, with retinal detachments resolving within 24 h. Subretinal fluid drainage was required in five of seven eyes, reflecting the relatively bullous nature of most detachments, whereas two eyes were successfully reattached without drainage. At both 1‐ and 6‐month follow‐ups, anatomical and functional improvements were maintained, demonstrating a 100% reattachment rate with no reported surgical complications or instances of redetachment.

**Conclusion:**

In this small, consecutive case series from a tertiary referral centre in Indonesia, SSB achieved favourable anatomical and functional outcomes at 6 months, with no redetachment or serious complications. The procedure was well tolerated, and in our resource‐limited setting, the material costs were lower than those of encircling buckling or vitrectomy based on local procurement prices, although we did not conduct a formal economic analysis. We therefore suggest that SSB remains a reasonable primary option for selected RRD cases, particularly in young, myopic patients with a single break. Larger, ideally multicentre studies are needed to confirm these observations and to explore generalisability to less experienced surgeons.

## 1. Introduction

Rhegmatogenous retinal detachment (RRD) is a sight‐threatening condition that requires immediate surgical intervention [1]. Surgical intervention is the preferred mode of treatment for RRD [2]. The scleral buckling (SB) or encircling buckle technique has been employed for the management of RRD. The encircling buckle technique has been shown to achieve good anatomical results, especially in cases with numerous breaks or extensive total retinal detachment. It involves the application of a silicone band around the entire eye circumference [3–6].

There is a trend toward using small‐gauge vitrectomy and wide‐angle viewing systems instead of SB in the management of RRD [7]. These approaches have recently gained popularity due to technological advancements and better ergonomics [8]. However, the gold standard treatment for primary RRD with a single break, minimal retinal detachment, lower quadrant detachments, adequate fundus visibility and clear lenses in young patients is still SB [9, 10].

The encircling buckle procedure has a success rate of > 94%. A 95% reattachment rate 20 years after surgery suggests long‐lasting effects, and the postoperative visual outcomes are generally favourable [11]. However, the encircling buckle procedure is associated with complications, such as reduced choroidal perfusion to the macula and optic nerve, risk of diplopia, refractive changes, progressive visual field loss, scleral erosion, anterior segment ischaemia, glaucoma and buckle erosion, intrusion, extrusion or migration [4, 12–14]. Nonetheless, the number of recorded complications is limited, and the procedure is considered highly safe [14].

Ophthalmologists use segmental scleral buckling (SSB) to reduce the risk of complications associated with the encircling buckle technique [3]. SSB involves gently indenting the sclera at the site of the retinal break and offering targeted support to the detached retina [5]. It may be placed obliquely, circumferentially or radially [3]. SSB offers significant advantages such as a much faster procedure, a decrease in the risk of diplopia and long‐term safety, as evidenced by the results of >15 years of follow‐up [6].

The initial success rate of SSB has been reported to be higher in some studies [6], although direct comparisons are lacking. However, the increased interest in vitrectomy has led to a decline in the use of SSB. This limited acceptance among ophthalmologists may be attributed to a lack of confidence in performing indirect ophthalmoscopy and uncertainty about SSB efficacy, as well as the technical challenges of the procedure [13]. In this descriptive case series, we present the anatomical and functional outcomes of SSB. While SSB has been described elsewhere, outcome data from Southeast Asia—particularly Indonesia—are scarce. This study therefore provides region‐specific, real‐world evidence.

## 2. Case Presentation

This retrospective study recruited seven patients who had undergone an SSB to correct RRD. The patients were treated by one surgeon (S.P.J.) at Sardjito General Hospital and YAP Eye Hospital. Inclusion criteria comprised patients presenting with primary RRD who underwent SSB, with up to two breaks within one quadrant, age ≥18 years and no prior vitreoretinal surgery. Exclusion criteria were defined as PVR grade B or higher, giant retinal tear, traumatic detachment or multiple breaks in more than one quadrant. All consecutive patients undergoing SSB for primary RRD between 2019 and 2022 were included; no eligible cases were excluded.

## 3. Baseline Characteristics

The baseline characteristic data were collected from electronic medical records: sex, age, duration of retinal detachment, condition of the fellow eye, preoperative best corrected visual acuity (BCVA), intraocular pressure (IOP), presence of relative afferent pupillary defect (RAPD), lens status, extent of retinal detachment, number and type of retinal breaks, and macular status. Additional perioperative and postoperative data were collected, such as the application of cryotherapy and/or subretinal fluid drainage (SRFD), intra‐ and postoperative complications and postoperative BCVA.

Each patient underwent a comprehensive ophthalmologic evaluation both pre‐ and postoperatively. This evaluation consisted of a dilated fundus examination, slit lamp biomicroscopy and tonometry. BCVA was measured using a Snellen chart and then converted to logMAR values for statistical analysis. The study was approved by the ethics committee (No. KE/FK/1094/EC/2024, 19 July 2024). Each participant provided informed consent before being enrolled in the study. SSB with a silicone band was performed. Cryotherapy and SRFD were performed if needed.

## 4. Surgical Details

All procedures were conducted under general anaesthesia. Our approach was characterised as ‘minimally invasive’ adaptation of conventional SSB, defined by three specific surgical parameters: (1) a limited conjunctival peritomy confined to the quadrant of the retinal break, avoiding 360° dissection; (2) selective, rather than routine, SFRD—performed only if the detachment was markedly bullous, could not be adequately flattened with buckle indentation and cryotherapy or if there was concern that the break would not close securely without drainage; and (3) avoidance of an encircling band, thereby preserving more normal ocular motility and reducing the risk of anterior segment ischaemia. In this small series, five of seven patients presented with relatively bullous macula‐off detachments, for which the senior surgeon judged SRFD to be the safer and more reliable option to achieve prompt retinal reattachment.

**Figure 1:**
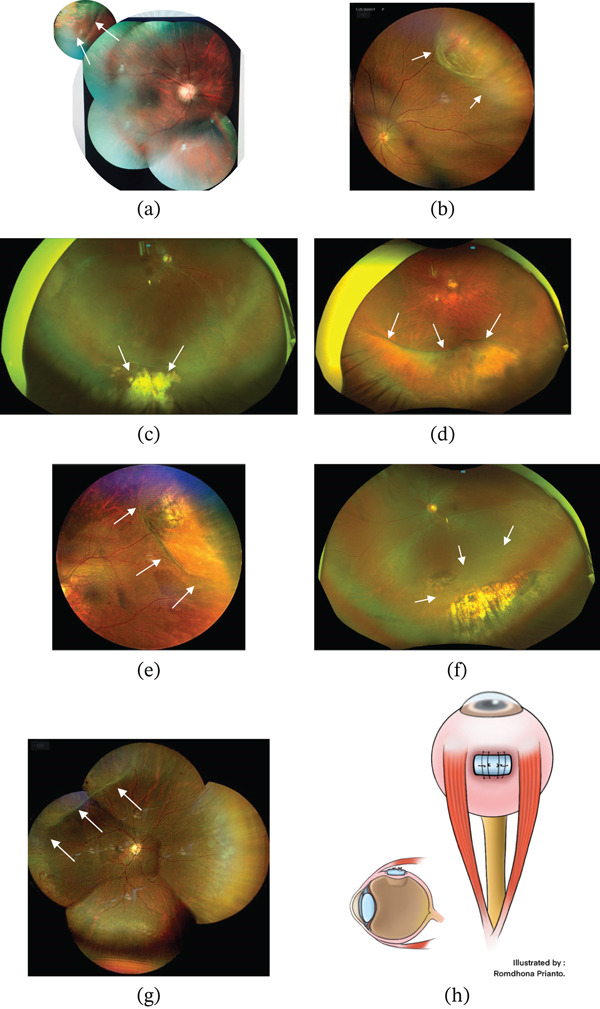
Fundus photograph after surgery and buckle position (arrow). (a) Patient 1 at superotemporal (arrow). (b) Patient 2 at superotemporal (arrow). (c) Patient 3 at inferior (arrow). (d) Patient 4 at inferior (arrow). (e) Patient 5 at superotemporal (arrow). (f) Patient 6 at inferotemporal (arrow). (g) Patient 7 at superotemporal (arrow). (h) SSB illustration.

The surgical approach (Figure 1h) involved creating a conjunctival peritomy creation at the site of SSB application, followed by dissection into the exposed quadrants. The rectus muscle was looped using a muscle hook and 2‐0 silk for eyeball fixation. The precise location of the retinal tear was confirmed on the sclera with the guidance of an indirect ophthalmoscope. If required, a 26‐gauge needle was used to externally drain the subretinal fluid, which was followed by cryopexy.

To secure the buckle, a 5.0‐mm silicone band (Style # 4050; Labtician Ophthalmics Inc., Oakville, ON, Canada) was sutured to the sclera circumferentially at the identified area using a 5.0 white‐braided polyester fibre suture (Mersilene; Ethicon Inc., Ohio, United States). An additional suture between the thread and the buckle was incorporated to prevent buckle migration, intrusion or extrusion. The buckle position and height were verified via indirect ophthalmoscopy. The Tenon′s capsule and conjunctiva were closed with 8.0 violet‐braided coated Vicryl (Vicryl; Ethicon Inc., Ohio, United States).

## 5. Outcomes

Anatomical and functional outcomes were analysed at 1‐ and 6‐month follow‐up visits. Anatomical success was defined as complete retinal reattachment following the initial procedure, including the use of pneumatic retinopexy if necessary. Functional success was defined as an improvement in BCVA of at least 0.2 logMAR from baseline. Changes in BCVA (*Δ* logMAR) were calculated as baseline logMAR minus follow‐up logMAR, such that positive values indicate improved vision and negative values indicate worsened vision.

The baseline characteristics of seven recruited patients who underwent SSB procedures are presented in Table 1. The cohort primarily consisted of young adults, with ages ranging from 27 to 50 years old. Their initial symptoms varied, the most common were blurred vision, photopsia (flashes of light) and field defects. Some patients also reported floaters. Baseline BCVA measurements obtained at the initial examination ranged from logMAR 0.8 to 2.3, indicating varying degrees of vision impairment. The IOP among the patients ranged from 10 to 18 mmHg. As the IOPs were within the normal limits, no patient exhibited immediate complications of acute pressure elevation.

**Table 1:** SSB patients′ profile.

Patient clinical condition	1 (OD) 37 years old female	2 (OS) 38 years old male	3 (OD) 27 years old male	4 (OD) 34 years old male	5 (OS) 29 years old male	6 (OS) 28 years old female	7 (OD) 50 years old male
Symptoms	Blurred vision Photopsia	Field defect	Blurred vision Field defect	Field defect Photopsia	Photopsia Field defect	Floaters	Field defect
Pre‐operative BCVA	logMAR 2.3	logMAR 2.0	logMAR 1.2	logMAR 1.5	logMAR 1.8	logMAR 0.8	logMAR 2.0
Postoperative BCVA (1 month POD)	Attached logMAR 1.5	Attached logMAR 1.3	Attached logMAR 1.5	Attached logMAR 1.5	Attached logMAR 0.6	Attached logMAR 0.3	Attached logMAR 1.3
Postoperative BCVA (6 months POD)	Attached logMAR 0.2	Attached logMAR 0.8	Attached logMAR 0.8	Attached logMAR 1.0	Attached logMAR 0.6	Attached logMAR 0.2	Attached logMAR 1.0
*Δ* logMAR (1 month POD)	0.8	0.7	−0.3	0	1.2	0.5	0.7
*Δ* logMAR (6 months POD)	2.1	1.2	0.4	0.5	1.2	0.6	1.0
IOP	10	14	18	15	11	13	13
RRD risk factors							
1. Refraction status	S −7.00	S −5.50	S −8.00	S −11.00	S −2.50	S +1.00 C −1.25	S −2.00
2. Lens status	Phakia	Phakia	Phakia	Phakia	Phakia	Phakia	Phakia
3. Previous viral retinitis, previous ocular procedure, trauma, hereditary vitreoretinopathy, RAPD and systemic disease	—	—	—	—	—	—	—
4. History of RRD in fellow eye	—	—	—	+	—	—	—

*Note:* Positive *Δ*logMAR denotes improvement in BCVA; negative *Δ*logMAR denotes worsening.

Abbreviations: BCVA, best corrected visual acuity; IOP, intraocular pressure; OD, oculus dextra; OS, oculus sinistra; POD, postoperative day; RAPD, relative afferent pupillary defect; RRD, rhegmatogenous retinal detachment.

At the 1‐month follow‐up, all patients exhibited favourable postsurgical outcomes, with successful retinal reattachment (Table 1). At the 6‐month follow‐up, the retinal reattachments were maintained, and the VA continued to improve and stabilised (Table 1). Furthermore, no complications such as reduced choroidal perfusion to the macula and optic nerve, diplopia, refractive changes, progressive visual field loss, scleral erosion, anterior segment ischaemia, glaucoma and buckle erosion, intrusion, extrusion or migration were reported. These findings suggest that SSB, when performed with good surgical technique and postoperative care, can be an effective surgical intervention for selected primary RRD cases.

The predominant risk factor for retinal detachment in these patients was high myopia (spherical equivalent values ranged from −2.00 to −11.00). Only one patient had hyperopia. All patients were phakic; they had not previously undergone lens surgery. Only one patient had a previous history of retinal detachment in the fellow eye. No patient reported a history of viral retinitis, previous ocular procedures, trauma or hereditary vitreoretinopathy.

Fundus photography findings and perioperative details for each eye are summarised in Table 2. Retinal breaks were predominantly located in the superotemporal or inferior regions of the retina. The breaks exhibited convex borders, corrugated surfaces and clear subretinal fluid in all the patients, indicating uncomplicated detachments. All seven eyes presented with macula‐off RRD. However, the individual retinal breaks themselves were relatively limited in extent, involving less than one to two clock hours in all cases. Undulation of bullae or folds with eye movement, another typical feature, was also observed in all patients. The retinal detachment resolved within 24 h after surgery, with satisfactory buckle position and height achieved (Figure 1a–g).

**Table 2:** Surgical details of patients undergoing segmental scleral buckling.

Patient clinical condition	1 (OD) 37 years old female	2 (OS) 38 years old male	3 (OD) 27 years old male	4 (OD) 34 years old male	5 (OS) 29 years old male	6 (OS) 28 years old female	7 (OD) 50 years old male
Fundus photography findings							
1. Location break	Superotemporal	Superotemporal	Inferior	Inferior	Superotemporal	Inferotemporal	Superotemporal
2. Extent of retinal break (clock hours)	Less than 1 clock hour	Less than 1 clock hour	Less than 1 clock hour	Less than 1 clock hour	Less than 1 clock hour	Less than 1 clock hour	Less than 1 clock hour
3. Number of breaks	1	1	1	1	2 (double small breaks)	1	1
4. Border	Convex	Convex	Convex	Convex	Convex	Convex	Convex
5. Surface	Corrugated	Corrugated	Corrugated	Corrugated	Corrugated	Corrugated	Corrugated
6. Subretinal fluid	Clear	Clear	Clear	Clear	Clear	Clear	Clear
7. Bullae/folds	Undulate with eye movement	Undulate with eye movement	Undulate with eye movement	Undulate with eye movement	Undulate with eye movement	Undulate with eye movement	Undulate with eye movement
8. Macula	Detached	Detached	Detached	Detached	Detached	Detached	Detached
9. PVR grading	A	A	A	A	A	A	A
10. SFRD intervention	Yes	No	No	Yes	Yes	Yes	Yes
11. Cryotherapy intervention	Yes	No	Yes	No	Yes	No	Yes
12. Gas intervention	No	No	No	No	No	No	No
Complications (reduced choroidal perfusion to both the macula and optic nerve, diplopia, refractive changes, progressive visual field loss, scleral erosion, anterior segment ischaemia, glaucoma, buckle erosion, intrusion, extrusion or migration)	—	—	—	—	—	—	—

*Note:* The values for extent refer to the circumferential extent of the retinal break (clock hours). All cases had extensive macula‐off rhegmatogenous retinal detachments.

Abbreviations: OD, oculus dextra; OS, oculus sinistra; PVR, proliferative vitreoretinopathy; SFRD, subretinal fluid drainage.

## 6. Discussion

The SB or encircling buckle has been the gold standard for RRD treatment for more than 60 years [10]. Custodis first introduced SBs for RRDs in 1949. Schepens performed the first documented SB procedure in the United States in 1951 [15]. It involves identifying retinal tears and treating them with cryopexy and silicone explants for external support [10]. The encircling buckle procedure uses an elastic sponge or band that extends over the entire retinal perimeter while draining the subretinal fluid. Meanwhile, SSB covers the region of the retinal break(s) without the need for drainage [16].

### 6.1. Comparative Analysis: Encircling Versus SSB

Encircling buckles are recommended for eyes with multiple retinal breaks, myopia, extensive vitreoretinal pathologies and proliferative vitreoretinopathy (PVR) graded B or higher. This procedure is preferred when retinal breaks are not visible, under the assumption that posterior breaks are rarely missed and minor anterior breaks typically manifest at the vitreous base. However, encircling SB can cause complications such as restricted extraocular muscle function and eyelid retraction. Thus, some practitioners adopt the SSB approach to minimise tissue manipulation [3].

While our study was not designed to compare SSB with encircling buckling, the favourable outcomes we observed suggest that SSB may be a reasonable alternative in selected patients. Potential advantages such as a shorter operating time, no need for subrectus dissection and preservation of more normal ocular motility have been described in the literature [17]. However, we caution that a direct comparative trial would be needed to confirm any advantage.

### 6.2. Surgical Technique: SSB With Silicone Band Explants

More recently, the foldable capsular buckle has been described as a novel SB variant for complex RRD [18]. While promising, this technique is not yet widely available in our setting. Our SSB approach using a silicone band remains a less costly option for primary RRD with single or multiple small breaks in one quadrant, based on local procurement prices, although we did not perform a formal economic analysis. In this case series, the SSB with silicone band explants was the main therapeutic approach. Pneumatic retinopexy was performed as needed. Because most of the patients with RRD at our health centre were young adults, SSB was considered a practical option. Although explants can be positioned circumferentially, radially or obliquely [3, 10], in our study, the band was placed circumferentially and sutured to the sclera′s outer surface. The buckle was placed based on Lincoff′s criteria in cases where retinal breaks were not evident [3].

In Banaee et al. study, the increased frequency of buckle extraction may limit the efficacy of segmental buckling with silicone sponges. Among the buckles removed, three were radially placed, and one was circumferentially placed. These were removed due to concerns related to motility. All other buckles were positioned circumferentially and were removed for cosmetic purposes [3]. In our study, a silicone band was selected over a silicone sponge to minimise the need for buckle removal. Furthermore, silicone bands are more accessible than silicone sponges in our country.

SSB has been frequently linked to complications such as haemorrhage, infection, double vision, buckle‐induced astigmatism, and buckle intrusion or extrusion [3, 5]. Thus, in our study, additional sutures were incorporated through the thread and band, to prevent buckle migration, intrusion or extrusion. At the 1‐month postoperative evaluation, a noticeable improvement in anatomical and functional outcomes was observed. The BCVA improved in all the patients, with a decrease in the logMAR values (e.g., from logMAR 2.3 to 1.5), and no redetachment was found. At the 6‐month follow‐up, no buckle migration, intrusion or extrusion was observed, and VA improvement or preservation was maintained.

In our case series of seven patients, we implemented SSB by meticulously choosing and compiling surgical experiences. In all cases, retinal reattachment was achieved successfully with no adverse effects. Our findings suggest good patient compliance, which may be related to the procedure′s short duration and perceived tolerability. However, we recognise that the perceived simplicity may reflect the senior surgeon′s experience; trainees or less experienced colleagues might find the technique more challenging, particularly during the initial learning curve. Ultimately, our case series demonstrates that, in our hands, SSB achieved good anatomical and functional visual outcomes for primary RRD in selected patients.

### 6.3. Limitations and Future Directions

Our study had some limitations. This was a small, consecutive case series from a single tertiary referral centre in Indonesia, performed by one experienced surgeon. We included all eligible patients without exclusions to minimise selection bias, but the lack of a comparison group (e.g., encircling buckling or vitrectomy) means we cannot draw comparative conclusions. Consequently, our findings may not be directly generalisable to other settings or to surgeons with different levels of experience. Nevertheless, we believe that the favourable outcomes justify further larger studies, particularly in low‐ and middle‐ income countries where vitrectomy is not always accessible.

Furthermore, variances in surgical duration, intraoperative haemorrhage and patient discomfort could not be objectively evaluated due to the absence of a control group. However, the relevance of our study findings extends to all procedures for treating segmental retinal detachment. We propose that our adapted SB technique may enhance the efficacy of SSB procedures, but adequately powered comparative studies are required to test this hypothesis against conventional SB.

## Author Contributions

Supanji Supanji: performed the surgeries, the study′s conception/design, postsurgical condition and critical revisions in intellectual content. Dewi Fathin Romdhoniyyah: drafting the manuscript, final revision of manuscript, reviewing of surgical content and postsurgery data. Hifdza Faza Felisha: project administrator and drafting the manuscript. Felicia Widyaputri: final revision of manuscript and reviewing surgical procedure. Tiara Putri Utami: patient care and postoperative follow‐up. Erin Arsianti: final revision of manuscript and reviewing surgical procedure. Mohammad Eko Prayogo: final revision of manuscript and reviewing surgical procedure. Firman Setya Wardhana: final revision of manuscript, reviewing surgical procedure and postoperative follow‐up. Muhammad Bayu Sasongko: final revision of manuscript, reviewing surgical procedure and postoperative follow‐up.

## Funding

No funding was received for this manuscript.

## Ethics Statement

The study conducted adhered to the ethical principles outlined in the 1964 Helsinki Declaration and was approved by the Medical Research Ethics Committee of the Faculty of Medicine, Gadjah Mada University (Number: KE/FK/1094/EC/2024, 19 July 2024). Written informed consent was obtained from each patient for all medical procedures, treatments and the publication of this paper.

## Consent

All the patients allowed personal data processing, and informed consent was obtained from all individual participants included in the study.

## Conflicts of Interest

The authors declare no conflicts of interest.

## Data Availability

This article contains all the relevant data that support the study′s findings, and any further inquiries can be directed to the corresponding author.
